# Determinants of an integrated public health approach: the implementation process of Greenland’s second public health program

**DOI:** 10.1186/s12889-018-6253-4

**Published:** 2018-12-07

**Authors:** Christine Ingemann, Barbara J. Regeer, Christina V. L. Larsen

**Affiliations:** 1 Centre for Public Health in Greenland, National Institute of Public Health, University of Southern Denmark, Copenhagen, Studiestraede 6, 1455 Copenhagen K, Denmark; 2 Athena Institute for Research on Innovation and Communication in Health and Life Sciences, VU University, Amsterdam, de Boelelaan 1085, 1081 HV Amsterdam, The Netherlands; 3 Greenland Centre for Health Research, University of Greenland, Nuuk, Postbox 1061, 3905 Nuussuaq, Greenland

**Keywords:** Public health program, Health promotion, Integrated approach, Determinants, Implementation process, Evaluation, Greenland, Inuit, Circumpolar health, Arctic

## Abstract

**Background:**

Greenland struggles with a high prevalence of smoking, alcohol and drug abuse. In response to the increasing need for preventive initiatives, the first public health program *Inuuneritta* was introduced in 2007. Internationally, frameworks focus primarily on the implementation of a single, well-described intervention or program. However, with the increasing need and emergence of more holistic, integrated approaches, a need for research investigating the process of policy implementation from launch to action arises. This paper aims to augment the empirical evidence on the implementation of integrated health promotion programs within a governmental setting using the case of Inuuneritta II. In this study, the constraining and enabling determinants of the implementation processes within and across levels and sectors were examined.

**Methods:**

Qualitative methods with a transdisciplinary approach were applied. Data collection consisted of six phases with different qualitative methods applied to gain a comprehensive overview and understanding of Inuuneritta II’s implementation process. These methods included: observations and focus group discussions at the community health worker (CHW) conference, telephone interviews, document analysis, and a workshop on results dissemination.

**Results:**

Enabling determinants influencing the implementation process of Inuuneritta II positively were high motivation among adopters, local prevention committees supporting community health workers, and the initiation of the central prevention committee. In contrast, constraining determinants were ambiguous program aims, high turnovers, siloed budgets and work environments, and an inconsistent and neglected central prevention committee.

**Conclusion:**

Inuuneritta II provided a substantial framework for an integrated health policy approach. However, having a holistic and comprehensive program enabling an integrated approach is not sufficient. Inuuneritta II’s integrated approach does not harmonise with the government’s inflexible organisational structure resulting in insufficient implementation.

**Electronic supplementary material:**

The online version of this article (10.1186/s12889-018-6253-4) contains supplementary material, which is available to authorized users.

## Background

Greenland has experienced the epidemiological transition from communicable to non-communicable diseases during the latter half of the twentieth century comparable to developing countries [1]. Major public health challenges include a high prevalence of smoking, dietary changes, alcohol and drug abuse as the top three identified risk factors that drive most death and disability in Greenland [2]. Based on population surveys in Greenland since 1993, the need for preventive initiatives addressing these risk factors increased [3, 4]. Thus, the first public health program *Inuuneritta* was implemented in 2007, followed by a second adapted program *Inuuneritta II* in 2013. *Inuuneritta*, Kalaallisut (Greenlandic) for ‘let’s have a good life’, aims to improve the quality of life of Greenlanders [3, 5]. This public health program can be perceived as a comprehensive intervention, which continuously is being implemented in Greenland. Most national health promotion efforts address health issues separately, whereas Inuuneritta II is a comprehensive public health program in terms of topics and target population. The program is described in a 30-page booklet both in Danish and Kalaallisut (Greenlandic). It was initiated by the Ministry of Health and focuses on the topic areas smoking, alcohol & hashish, diet and physical activity, which are equally described [5]. The program includes both health promotion and prevention activities, such as national campaigns promoting increased physical activity at the workplace and alcohol legislation preventing excessive intake of alcohol [6, 7]. These two terms are applied interchangeably in this paper, since many activities in Inuuneritta II can be categorised under both terms.

Internationally, frameworks focus primarily on the implementation of a single, well-described intervention or program focusing on for example only smoking among adolescents [8–12]. The effectiveness of such interventions and programs in health promotion can be influenced by several factors related to the development, implementation or sustainability of a program [9, 13, 14]. Local ownership of a program is one of these influencing factors [15, 16]. Still, existing evidence supports that health promotion can be a cost-effective way to improve health [10], and is recognised to enable people to increase control over and improve their health [17, 18].

The Ottawa Charter for Health Promotion points out that ‘health promotion goes beyond health care’, which indicates that all sectors and levels need to be involved and have to collaborate [17]. Inuuneritta was initially based on the Ottawa Charter. A comprehensive program like Inuuneritta II provides the opportunity for an integrated approach, where the involvement and collaboration of actors within and across sectors is enabled. Such an integrated approach makes the implementation process complex. The success or failure of a program is largely determined by the implementation process [19]. Hendriks et al. (2013) point out how most research describing integrated policy approaches are set within organisational, not governmental, settings [20]. Research investigating the process of policy implementation from launch to action represents a major knowledge gap.

The aim of the study was to investigate the constraining and facilitating determinants of the implementation process of the public health program Inuuneritta II within and across levels and sectors.

### Organisational structures in Greenland

Despite Greenland’s large geographical size, it is the least densely populated country in the world with a total population of 55,877 [21]. Ninety percent of the population are ethnic Greenlanders (Inuit). The population is scattered across 16 small towns and approximately 60 communities which are all situated on a narrow coastal strip. In the beginning of 2018, 86.8% of the population lived in urban areas [21]. The capital Nuuk has 17,796 inhabitants, the second largest town Sisimiut has 5491 and communities have between 500 and less than 50 inhabitants. There are no roads connecting communities. The majority of the population (92%) lives on the west coast. Countrywide, there are marked socioeconomic and infrastructural differences between towns and communities, where the communities in the East and extreme North are poorer and less developed than the rest of the country [22].

**Fig. 1:**
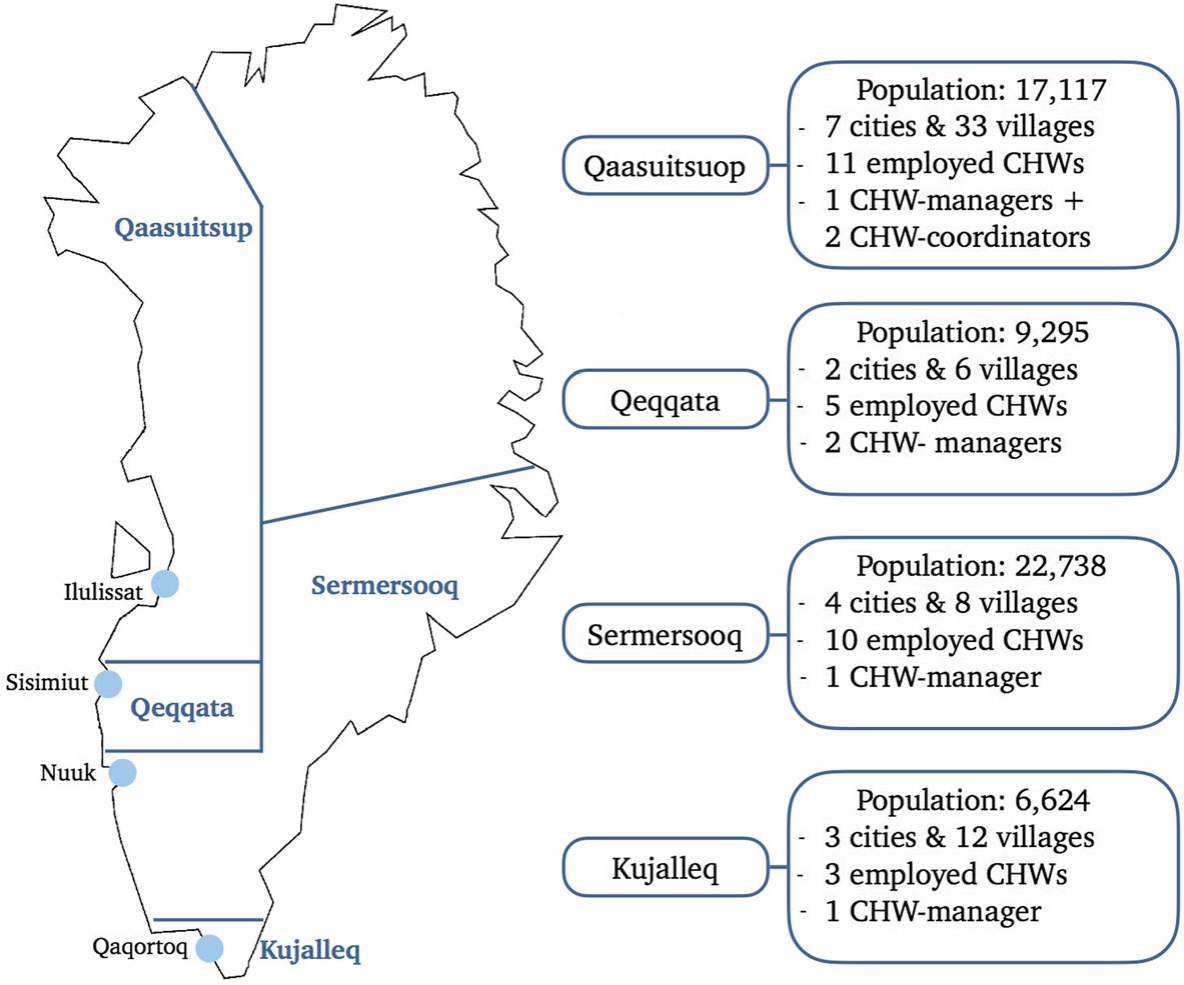
Greenland’s municipalities (status 2017)

**Fig. 2:**
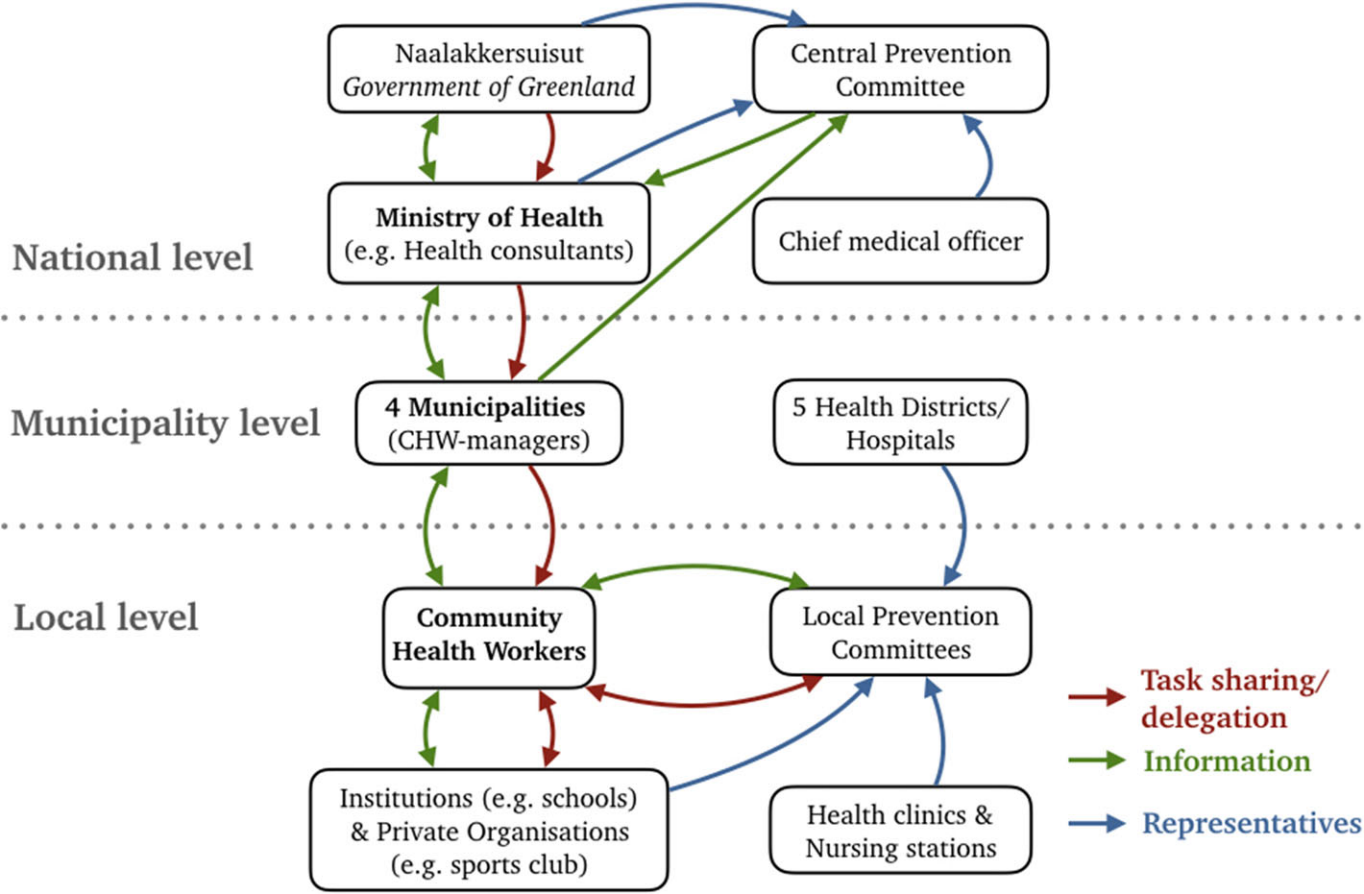
Stakeholders & organisational structures

Greenland is a former Danish colony, which gained Home Rule in 1979, and it has roughly adopted the Danish welfare-state model. The national language is Kalaallisut (Greenlandic), but in general Danish is taught in schools and in bigger cities Danish is often the primary working language. At the time of the study, Greenland was divided into four municipalities (5 municipalities since 2018). In Fig. 1, a small map of Greenland and its municipalities is provided, visualising the differences in population as well as the number of community health workers (CHWs) and managers (CHW-managers) employed at the point of data collection [23]. The Greenlandic government has decentralised preventive health services to the four municipalities [2, 24]. The overall responsibility for the implementation of Inuuneritta lays with the Ministry of Health. However, in practice, the preventive and promotion work of Inuuneritta II requires efforts at all levels (vertically) and across sectors (horizontally). With Inuuneritta II the strategic initiative a central prevention committee (CPC) was established, which forms a central counterpart to the local prevention committees (LPCs). The members consist of the permanent secretary of different ministries such as health, education, finance etc., as well as representatives of the national police, the chief medical officer, and the municipality association. The CPC is responsible for the coordination of the cross-disciplinary cooperation on all structural levels and securing anchorage of Inuuneritta II on the highest political level. Figure 2 provides an overview of the main stakeholder groups and their defined relations within the program [4, 5, 25].

## Methods

### Conceptual framework

The conceptual framework was based on implementation and system theories. Systems thinking highlights entanglements and interdependencies of a system [26]. Many scholars have emphasised how systems thinking is a way to understand the interaction and influence of elements within a system [27–31]. The determinants’ interlinkages and influence on various actors necessitate a multilevel and multi-actor view – an essential component for systems thinking.

A determinants framework with a systems thinking approach by Greenhalgh et al. (2004) guided both the data collection and analysis. The authors developed a comprehensive model of the various determinants influencing implementation processes [28]. A model aiming to provide guidance in assessing complex situations of the diffusion of innovations in organisations [28, 32]. In order to limit the complexity of the study, only three of the model’s nine categories of determinants were selected and applied to the context of this study; 1) the intervention (compatibility, complexity, reinvention), 2) the adopter (motivation, ability), and 3) implementation and routinisation (adaptiveness, leadership & feedback, interorganisational networks & communication, human resources & funding) [28]. The three categories of determinants were chosen based on thorough discussions with key stakeholders and authors prior to data collection. The determinants within the *intervention* category describe for example how decreased *complexity* of an intervention enables implementation. *Adopters* of Inuuneritta are stakeholders involved in the implementation, whose motivation and ability are crucial to the process. The determinants of the *implementation and routinisation* category are closely linked and influence each other.

Such a combined framework of systems thinking and implementation determinants assisted the identification and understanding of how and where to improve and strengthen the organisational structures related to Inuuneritta II [26, 27].

### Qualitative methods

**Fig. 3:**
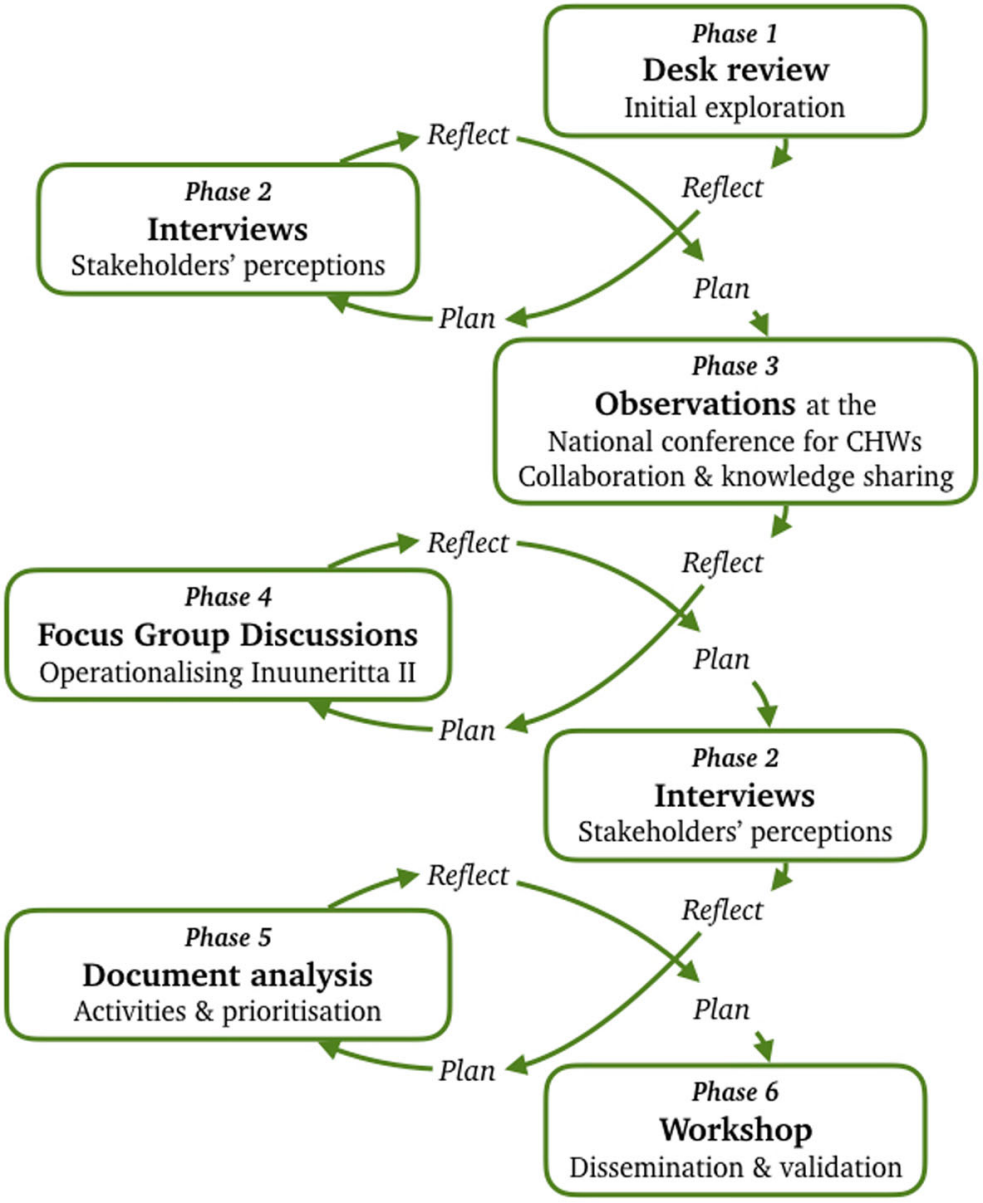
Flow of data collection

Qualitative methods with a transdisciplinary approach were applied in all six phases of data collection (see Fig. 3), thereby securing the involvement of relevant academic and non-academic stakeholders in the research, and the identification of interactions and relationships across stakeholders. The involvement of various academic and non-academic stakeholders, who hold multiple perspectives generates ‘socially robust knowledge’ [33, 34] and is considered to increase the validity of the findings [35]. Furthermore, an emerging design was applied with reflective feedback-loops ensuring the relevance of each phase of data collection (see Fig. 3).

### Data collection

Data collection consisted of six phases with different qualitative methods applied in order to gain a comprehensive overview and understanding of Inuuneritta II’s implementation process (Table 1). This included 11 interviews, observations, 3 focus group discussions (FGDs), document analysis of 61 documents (see more Table 1) and a dissemination workshop conducted by the first and last author of this paper. Due to Greenland’s small population size, all stakeholders were contacted and invited to participate in the study, thereby making the pilot testing of interviews and workshop guides inaccessible. All phases contributed to the identification of the enabling and constraining determinants in the implementation process. Participants were informed about the purpose of the study prior to participation and gave informed consents.

**Table 1:** Data Collection

Phases & method	Aim	Participants
1. Desk review	Initial exploration	Scientific and grey literature
2. Semi-structured telephone interviews (45 min)	Key stakeholders’ perceptions of Inuuneritta II	2 CHWs
		3 CHW-managers
		6 Health consultants
3. Observations at national conference	Collaboration & knowledge sharing	24 CHWs
(3 days in Nuuk)		2 CHW-managers
		8 Health consultants
		6 Representatives from the Hospital Health Prevention Programs
4. Focus Group Discussions	Operationalising Inuuneritta II	Homogenous groups:
(part of the national conference, 3 h)		1) 23 CHWs + 2 CHW-managers (further divided into groups of 5)
		2) 5 Health consultants + 4 Representatives from the Hospital (further divided into groups of 3)
5. Document analysis	Activities and prioritisation of Inuuneritta II across levels	61 documents were collected:
		Local annual reports & action-plans
		CPC meeting agendas & minutes
		Municipal policies & strategies
		National strategies for topic areas
6. Workshop	Dissemination & validation	1) Dissemination and FGDs within the Ministry of Health - 12 Health consultants
(3 days in Nuuk)		2) Dissemination and feedback discussions with the central prevention committee

#### Semi-structured interviews

The semi-structured interviews (phase 2) were conducted in Danish over telephone by the primary researcher situated in Denmark with a pre-developed interview-guide (see Additional file 1). Interview questions were developed based on the conceptual framework, and touched upon the following topics: i) topic areas and target groups prioritised, seizing and challenges with the program on municipal and national level; ii) own, colleagues’ and leaders’ perceptions; iii) collaboration within and across levels and sectors; iv) challenges and suggestions for improvement of the implementation process.

#### Observations & focus group discussions at the national conference

At the national CHW conference in Nuuk (Greenland) the researchers observed participants (phase 3) and held two homogenous FGDs (phase 4), which were held in both Danish and Kalaallisut using professional simultaneous translators. The overall topics addressed were, i) where do you get your knowledge from, ii) what are the major health challenges you see in your work, iii) assess Inuuneritta II’s topic areas and aims, and iv) write a postcard to a stakeholder you would like to ask for help.

#### Workshop

The workshop (phase 6) was conducted in Nuuk (Greenland), and contributed to the validation of the study’s findings, the concluding recommendations of the study and it ensured the dissemination of results. Here, one FGD was held after the dissemination of the results. Participants discussed the questions: i) what needs to change so that we can build on the good experiences, ii) what should the next Inuuneritta look like, and iii) what needs to be in place to facilitate the implementation of the program’s last two years. Only national stakeholders participated, since local stakeholders (CHWs, CHW-managers) were not able to attend.

### Data analysis

During data collection, interviews were recorded and transcribed, and notes were taken during the conference, FGDs and workshop. Kalaallisut material was translated verbatim to Danish. The notes and transcripts were coded in the qualitative data analysis program NVivo (version 11) by the primary researcher. Here, both deductive and inductive coding was applied [36], where the conceptual framework gave the primary coding-frame, and emerging themes were added. Documents were analysed by transferring data into excel sheets in order to gain an overview of the extracted information. Quotes in this paper have been translated verbatim from Danish into English.

## Results

The identified determinants of the implementation process of Inuuneritta II are described in detail below and are supported by quotes of participants from interviews, FGDs and the workshop. The results are presented based on the three categories of determinants identified through the conceptual framework that guided the data collection and analysis.

### The intervention - determinants related to Inuuneritta II

The enabling and constraining determinants related to Inuuneritta II as the *intervention* regarding its *compatibility, complexity and reinvention* are summarized in Table 2. The four topic areas of Inuuneritta II are to some extent *compatible* with community health workers’ (CHWs) perceptions of health issues they experienced their communities to struggle with. This came forward during the national conference and focus group discussions. However, participants agreed that mental health or resilient citizens is a lacking topic in the program. *‘In Inuuneritta there is written too much about our problems instead of how we can solve these problems in terms of prevention’* (CHW 1). Descriptions like these by participants indicate that the program is not *compatible* with the CHWs’ needs.

**Table 2:** The Intervention

Enabling determinants	Constraining determinants
• The four topic areas are to some extent compatible with CHWs’ experiences of health issues present in their communities.	• Inuuneritta II does not include mental health or resilient citizens as a topic area, it only refers to the National Strategy for Preventing Suicide.
• The program’s operation-based schedule provided a guideline and attention, and decreased the program’s complexity.	• The program descriptions are not compatible with CHWs needs, who criticise it to be too problem-focused instead of providing hands-on guidance.
	• Action-plans developed by health consultants are again too problem-focused and rarely applied.
• The few specific aims in the program decrease its complexity, and in some cases unspecific aims can leave room for reinvention and adaptation to the local context.	• Most of Inuuneritta II’s aims are too broad and ambiguous.

Health consultants (HCs) at the national level described that the program’s operation-based schedule for initiation of the topic areas, had given each topic area successively additional attention (*complexity*) thereby enabling implementation. National action-plans were developed as a supplement for each topic area in Inuuneritta II. The general aim of these was to provide an overview of the existing and planned national initiatives in Greenland. Prior to publication, action-plans were forwarded to relevant stakeholders such as municipalities, ministries or researchers in order to receive feedback. One HC described that the action-plans *‘…are a starting point for collaboration… [and] can be used as a guide: these are the activities we will focus on…’* (HC 1) (*complexity*). However, others pointed out how the many hours of work put into developing action-plans are wasteful compared to the eventually rare use of them. Based on the performed document analysis, the content of the action-plans is mainly descriptive providing no guidance to CHWs.

For the four aims of each topic area, FGD-participants defined key-stakeholders and action-points. Participants reported that this was the first time for them to be assessing the aims in detail. It quickly became clear that the aims were too vague and unspecific. Based on participants statements, aims which are not specific can on the one hand leave room for *reinvention* and adaptation to the local context, on the other it can make CHWs’ work content and responsibility unclear (*complexity*).

### The adopter

A summary of the enabling and constraining determinants of the adopter are shortly described in Table 3. *Adopters* of Inuuneritta II are stakeholders involved in the implementation process, determinants within this category are motivation and ability. Adopters are HCs, CHW-managers and CHWs. HCs and CHWs expressed and showed great *motivation* for contributing to public health and interest to work with Inuuneritta II’s topic areas. *‘I experience that they [CHWs] actually take it [working with Inuuneritta] very serious. …They take the responsibility [for it]’* (HC 3).

**Table 3:** The Adopter

Enabling determinants	Constraining determinants
• Adopters are greatly motivated to work with health promotion.	• Many CHWs feel alone and overwhelmed with their work tasks.
• Health consultants have relevant educational knowledge and experience.	• Health consultants lack expertise for evaluating initiatives.
	• As a newly employed health consultant it is challenging to acquire the necessary background knowledge of the program.
• CHWs use the knowledge resources available to them (e.g. LPC).	• CHW-managers typically focus on treatment instead of health promotion. Thereby guidance to CHWs can be confusing.
	• CHWs have in general a low level of education, which rarely relate to health promotion.

HCs generally have a background within health and have work experience from the municipality level (*ability*). The CHW-managers are most often social workers with no educational background in health promotion working under the municipal administration *Social and Family affairs*. This can constrain implementation, since CHW-managers cannot regularly provide the necessary guidance to CHWs (*ability*). CHWs described that it is difficult to work with a health promotion mindset, when your manager focuses on treatment. Next to Inuuneritta, CHWs also have many other tasks to take care of locally, such as one CHW also being a social worker. The educational level of CHWs is typically low, especially within the area of health and social issues. CHWs showed a great interest in improving their own competencies (*ability*), but only few have received additional courses relating to the topic areas of Inuuneritta II.

Adopters recognised in interviews to lack competencies (*ability*) in evaluating initiatives: *‘If I should really look at an area where I really really am in need for education, then it is evaluation’* (HC 2). Furthermore, when starting as an HC at the Ministry of Health, interviewees described the challenge of acquiring the necessary background knowledge of Inuuneritta II. Despite having access to reports and experienced colleagues’ knowledge, it remains time-consuming and important knowledge gets lost due to lack of appropriate hand-overs.

On average, a single CHW is alone with the task to provide health promotion to one city and three or more distant surrounding communities, this is an overwhelming task for CHWs and by some experienced to be too big of a responsibility to carry alone (*motivation* + *ability*). One of CHWs’ main strength, however, is making use of the knowledge resources available to them such as the local prevention committees (LPCs) (*ability*).

### Implementation & routinisation of the intervention

In Table 4 the enabling and constraining determinants of the implementation and routinisation of Inuuneritta II are summarized. The determinants of the *implementation and routinisation* category include *adaptiveness, leadership, feedback, interorganisational networks & communication, human resources & funding.*

**Table 4:** Implementation and Routinisation

Enabling determinants	Constraining determinants
• Adopters across levels agree that they have a shared responsibility for the implementation of Inuuneritta II	• “When everyone has the responsibility, then no one has it.” (HC4)
	• Overall operational coordination lies with the Ministry of Health
	• Budget is divided into topic areas
• Initiation of the central prevention committee (CPC) ensuring intersectoral collaboration	• CPC meetings are inconsistent in context and participation of members
	• Collaboration across ministries has not been politically prioritised neither within ministries
	• The CPC does not collaborate with nor monitor LPCs
• Public-private partnerships have been initiated by health consultants	• Stakeholders of public-private partnerships are not held accountable
• Inuuneritta is part of local health policies	• High turnover of employees in the Ministry of Health
• The few well-functioning local prevention committees (LPC) support the work of CHWs	• CHWs lack a coordinating body
	• Language barriers between CHWs and HCs constrain vertical communication
	• Lack of human resources in the Ministry of Health and locally in municipalities

An enabling precondition for the enhancement of collaboration across levels is the fact that participants of the study agreed that the responsibility for the implementation of Inuuneritta II lies with all stakeholders from national, municipal and local levels (*interorganisational networks & communication*). A HC described in an interview that the responsibility of Inuuneritta II’s implementation is *‘…50/50 between the Ministry of Health and the municipalities. We have a shared responsibility. We [health consultants] depend on them [CHWs]’* (HC 4). However, this is also a constraining determinant as pointed out by a HC in the final workshop: *‘when everyone has the responsibility, then no one has it’* (HC 5).

Vertical collaboration between national, municipality and local level was reported to be challenging by several participants (*interorganisational networks & communication*). For HCs, it can be difficult to motivate CHWs to collaborate on different initiatives due to typically limited available financial and human resources in municipalities (*human resource & funding)*. Participants wish to prevent top-down approaches in the collaboration; however, the vast geographical distances between cities and communities often means that HCs do not know CHWs’ context and work environment. Furthermore, cultural differences and language barriers can lead to misunderstandings in work practice. Regarding the language barrier, most of the HCs, who are only fluent in Danish, rely on their colleagues fluent in Kalaallisut to function as a link to initiate collaboration. These challenges present possible explanations for the rare contact between CHWs and HCs. A CHW described: *‘I am all alone here in my little world. …But they [HCs] usually send me an e-mail, when they are having a [national] campaign’* (CHW 2).

The overall operational coordination of Inuuneritta II lies officially with the Ministry of Health, where the allocated national budget is separated into Inuuneritta II’s topic areas, just as the HCs are recruited for each separate topic area (*human resource & funding*); alcohol & hashish 195,000$, smoking 69,000$, physical activity 70,000$, diet 86,000$ [37]. The document analysis showed that high turnover of employees in the Ministry of Health has occurred during Inuuneritta II’s implementation (*human resources*), which has been a constraining determinant. Interviewees described how turnover of HCs delayed initiatives, and turnover of leaders resulted in unclear direction and guidance (*leadership*).

The document analysis showed that each municipality refers to Inuuneritta in their own health policies (*adaptiveness*). Since health promotion on the local level is decentralised to municipalities, it is the responsibility of each municipality to allocate budgets for their health promotion and prevention work. This means, in practice, budget allocation varies between municipalities and throughout time (*human resource & funding*). A CHW described: *‘We are only two [CHWs] and we coordinate everything ourselves and we deal with everything ourselves and we also do not have any budget, so we always need to find some sponsors’* (CHW 1).

#### Interorganisational network & communication

With Inuuneritta II, two strategic initiatives supporting *interorganisational communication and networks* were introduced. First, the establishment of a central prevention committee (CPC) as a counterpart to the local prevention committees (LPCs), and secondly the initiation of health collaboration agreements between the Ministry of Health and private companies or associations.

The majority of the study’s participants noted that no overall body coordinates and administrates the health prevention and promotion work in Greenland. CHWs develop annual strategies for the cities and communities they are responsible for together with their local CHW-manager or LPCs with the aim to develop locally relevant initiatives (*adaptiveness*). The conducted interviews showed that the efficacy of the LPC varies among the cities in Greenland (*interorganisational network*). The annual strategies are expected to be sent to the national level for coordination; however, this rarely happens and if it does, no *feedback* is provided.

Since the establishment of the CPC, meetings have been held twice a year with infrequent attendance of members or typically not orientated representatives. The minutes indicate that the meeting content is: repetitive of informing participants of the committee’s purpose, rarely discussing Inuuneritta II’s topic areas, and lacking action points as a result from the meeting. A HC described the committees’ ineffectiveness to be due to the lack of political prioritisation and supportive resources. Furthermore, cross-disciplinary collaboration on the national level was described by a HC to be *‘non-existing regarding Inuuneritta’* (HC 3). Another described: *‘I experience it [the collaboration with other ministries] to be very difficult and sparse’* (HC 1). However, absent collaboration should not only be prioritised in other departments, but also in the Ministry of Health. These factors withhold the initiation of the CPC to be an enabling determinant for Inuuneritta II’s implementation.

Different public-private partnerships across organisations and associations have been initiated to increase cross-disciplinary collaboration, develop *interorganizational networks* and to increase the focus on health promotion in all areas. Some agreements have led to small success, such as specific weekdays where fish is on sale in supermarkets. However, in general the agreements were described to be ineffective since the stakeholders of the agreement are not held accountable: *‘…it’s not something that is binding’* (HC 2).

## Discussion

This study investigated the constraining and facilitating determinants of the implementation process of the public health program Inuuneritta II within and across levels and sectors of the government setting by applying qualitative and transdisciplinary methods.

Enabling determinants influencing the implementation process of Inuuneritta II positively were high motivation among adopters, local prevention committees supporting community health workers (CHWs), and the initiation of the central prevention committee. These enabling determinants can be jeopardised, when multiple constraining determinants continue to make the health promotion and prevention work of adopters (e.g. CHWs) burdensome. Some of these constraining determinants were ambiguous program aims, high turnovers, siloed budgets and work environments, and an inconsistent and neglected central prevention committee. The political context most often constrains health promotion initiatives, since improving health requires a long-term process and politicians focus on their short-term election cycles [9, 20]. This is also the case for Inuuneritta II, where the lack of political prioritisation has constrained implementation of Inuuneritta II across sectors (the poor performance of the CPC), across levels (lack of guidance and coordination) and within sectors (constrained program resources).

### Moving from silos to integration

Berkeley and Springett (2006) point out how in health promotion health issues should not be addressed separately, but rather in their full complexity; using a holistic approach, the several activities happening simultaneously are recognised and acted upon [9]. This is an aspect enabled by the Inuuneritta II program, which is a comprehensive and holistic program that provides a starting point for horizontal and vertical integration. The program itself, the CPC, and LPC enable an integrated approach, other organisational structures constrain this approach due to human and financial resources and operational responsibility being earmarked and organised in silos: 1) HCs are recruited by topic areas, 2) the overall operational responsibility lays with the Ministry of Health, 3) Inuuneritta II’s budget is placed under the Ministry of Health, and 4) the budget is divided into topic areas. These organisational structures make it difficult for HCs to transcend barriers and to work holistically and cross-disciplinary with Inuuneritta II. Furthermore, the separation of Inuuneritta II’s budget and the lack of financial resources for CPC, LPC and CHWs’ work constrains their efforts for Inuuneritta II. According to O’Flynn (2016), challenges ‘associated with the operation of the machinery of government itself’ are one of the reasons for public policy programs failing to translate ambitious headlines into on-the-ground action [38]. Recruiting HCs by topic area is relevant to ensure expertise; however, in order to enable an integrated approach this paper suggests that the Inuuneritta II program moves from focusing on topic areas to target groups, as well as increasing collaboration between HCs across topic areas.

### Work environment of Inuuneritta II

In the review of empirical studies on program sustainability by Pluye et al. (2004), common aspects in implementation and sustainability of programs are described [13]. One of these is the investment of adequate resources to complete activities (staff, funding, equipment, training) [13]. Resources for health promotion initiatives in Inuuneritta II are time constrained and limited financially, due to a scarce number of HCs and CHWs assigned to cover initiatives of the whole program: i) the high turnover delays initiatives on the national level, ii) the treatment focus on the municipality level distracts from Inuuneritta II’s aims, and iii) the low educational level of CHWs and overwhelming responsibilities limits activities on the local level. Increasing financial resources is often challenging; however, a change in the work culture could be enabling to retain employees and secure stability in the initiatives of the program. This can be done by enabling self-organising structures within the Ministry of Health in order to encourage creativity and social mixing [7], by ensuring transparent communication between adopters and leaders [13, 39], and by providing pathways for cross-talk and promoting knowledge exchange between employees [7].

Next to differences in bureaucratic work culture, there are also contextual differences based on geographical context and cultural background of adopters, which continuously influences the implementation processes within a complex system [29]. In Greenland, many of the positions at central level are occupied by often temporary workers from Denmark. Whereas positions at municipal and local level are more often occupied by Greenlanders. Differences in mother tongue and cultural background can lead to misunderstandings in communication and collaboration between Danish and Greenlandic workers within and across levels, which also was reported by interviewees. Due to the disproportional living and social standards existing in Greenland, discrepancies can also occur between Greenlandic workers from different social income classes. For example, having lived and received the opportunity to attend higher education levels in Nuuk, the capital city of Greenland, or in Denmark, can disconnect a person’s awareness for the living and social conditions existing in Greenland outside Nuuk and particularly in remote communities.

### Horizontal collaboration & vertical coordination

Implementing the public health program Inuuneritta II does not only entail one implementation process. Each new tool that Inuuneritta II introduces to the system, such as the CPC, must go through its own implementation process, which makes the implementation more complex. It is evident that the CPC has not received the undivided attention it required for establishment. It is failing to set an integrative agenda and engage members, which is according to interview participants attributed to the lack of leadership and political prioritisation. Pluye et al. (2004) describe in their review on sustainable public health programs how standardisation of activities through state-level rule will increase sustainability by giving ‘rise to more durable standardised routines’ [13]. This could be enforced for CPC meetings, where the general agenda, responsibilities and obliged presence of members is standardised and politically ensured.

The initiation of the central prevention committee (CPC) aimed to integrate Inuuneritta II across sectors and levels. This refers to a more holistic *whole-of-government* approach, which seeks to eliminate conditions of different policies undermining each other through horizontal (across sectors) and vertical (across levels) integration [40]. In vertical and horizontal integration, efforts over multiple system levels and across sectors are combined and coordinated [41]. This requires attention to the systems’ structures and processes [41]. A change in behaviour of adopters and the system is needed for them to set health and Inuuneritta II on their agendas and collaborate across sectors and levels [7, 42]. Here, the CPC provides a great forum to do so, but as this study shows coordinating vertical and horizontal health integration is highly complex and challenging. This is something widely discussed within international literature on *Health in All Policies* (HiAP) [43]. Greer and Lillvis (2014) describe how the coordination and durability of the HiAP approach does not only depend on a strong political leadership, but also on bureaucratic change and indirect strategies [20, 43]. As Clavier puts it, ‘formulating and agreeing on HiAP is all but the end of the process’ [44]. The CPC should not only be politically prioritised, but the governmental organisation needs to move away from silo-minded work paths towards cross-collaboration supported by innovative environments and leadership [45].

The local counterpart to the CPC are the LPCs whose efficacy varies among the cities in Greenland. The well-functioning LPCs provide CHWs with the sought knowledge or guidance they lack, and have a great potential to contribute to local horizontal integration of and community involvement in Inuuneritta II [42]. Here, directions and guidance from the CPC would enable LPCs to have a greater impact, when common vision and leadership horizontally and vertically are given. Vertical guidance was also found to be an enabling determinant in Guglielmin et al.’s (2017) scoping review on local HiAP implementation [46]. The authors describe how national leadership facilitates HiAP implementation by guiding municipalities [46]. The present findings show that this is not occurring in the case of Inuuneritta II due to the CPCs ineffectiveness.

### Future research & perspectives

Several stakeholders in research and policy promote integrated policy approaches to address and solve complex, real-world problems (e.g. HiAP). However, evidence on integrated policy approaches’ effects and implementation are scarce [47]. This highlights the need for studying integrated approaches in organisations going beyond theory. This study shows that an integrated program design does not automatically lead to horizontal and vertical collaboration in governmental settings. More research providing insights into the conditions for and implementation of successful integrated approaches within governmental settings is needed with emphasis on monitoring what works for whom under which circumstances. Furthermore, this study confirms the importance of following the implementation process of programs closely in order to ensure their sustainability [13]. Involving and enforcing the local level vertically and enabling collaboration across sectors, horizontally.

One way to enable implementation and secure program sustainability, is to promote adopters to gain ownership of the program [15, 16, 46]. Ownership of the program enables cross-disciplinary collaborations and secures adopters’ motivation to work with Inuuneritta II. In order to create ownership of the program and to ensure that the socio-environmental context is taken into consideration, key-stakeholders need to be involved in the planning of health promotion programs, which scholars have found to be an effective health promotion strategy [13, 16, 28, 42]. Pluye et al. (2004) describe that ‘sustaining programs in communities requires a collaboration [with stakeholders] from the beginning’ [13]. A collaborative discussion between adopters on the intervention’s meaning, the system’s structure and its power relations will stimulate ‘the creation of common ground and help create sustainable changes and innovations’ [29]. Furthermore, it will initiate a shared learning process, which will create ownership of and motivation to adopt the intervention [29, 48]. However, Berkeley and Springett (2006) point out the persistent challenge on how due to cultural differences between communities and powerful stakeholders, on many occasions, communities have difficulties in gaining an ‘equal seat at the table of partnership’ [9].

### Strengths & limitations of the study

The following limitations of the present study have been identified by the authors. First, one of the four CHW-managers could not be interviewed at the point of data collection, due to restructuring and elections in the municipality. Secondly, representatives from the LPCs nor citizens in Greenland were not included due to the scope of the study. These stakeholders would likely have been able to provide valuable insights to the health promotion and prevention efforts on national and local level. Furthermore, the conducted interviews were only held in Danish, which for some participants was the second language, and thereby may have led to misunderstandings or limited responses.

A major strength of this study is the triangulation of methods in data collection and the various academic and non-academic perspectives of key-stakeholders included, which is expected to have generated ‘socially robust knowledge’. These aspects strengthened the validity and credibility of the present study [49, 50]. The researchers were in direct contact with almost all community health workers and health consultants employed at the time of data collection, whereby a high level of data saturation was reached. Another strength of the study is that participants were able to communicate in their preferred language, Danish or Kalaallisut (Greenlandic), at the conference, on e-mail, in the focus groups discussions and workshop. Finally, data was collected and analysed by two researchers, which increases the internal observer reliability [51] and strengthening the present study.

## Conclusion

The Greenlandic public health program Inuuneritta II has provided a substantial framework for an integrated health policy approach. It comprises all public health focus areas in a single program, and initiated trans-sectoral collaboration through the central prevention committee and the local prevention committees. However, having a holistic and comprehensive program framing and initiating an integrated approach is not sufficient. Inuuneritta II’s framework does not harmonise with the government’s inflexible organisational structure resulting in insufficient implementation. In other words, the siloed structure of the governmental organisation constrains Inuuneritta II to have the full effect that it potentially could. For this reason, the established governmental organisation, lack of political prioritisation, lack of direction from leaders, and contextual differences in work cultures need to be overcome. Furthermore, local involvement is necessary in order to create ownership among adopters and thereby ensuring sustainability of Inuuneritta II. Adopters involvement and cross-sectoral collaboration should be attained at the beginning of the program rather than at the point of implementation. An aspect attainable for the upcoming third program due to Inuuneritta II’s established framework and lessons learned.

## Additional file

Additional file 1: **Table S1.** “Interview guides of the conducted semi-structured interviews” provides the interview questions used in the semi-structured interviews with CHWs and HCs, which were translated verbatim from Danish to English for the purpose of publication. (PDF 134 kb)

## Abbreviations

CHW: Community health worker
CPC: Central prevention committee
FGD: Focus group discussion
HC: Health consultant
HiAP: Health in All Policies
LPC: Local prevention committee
