# Supplementary material for: Determinants of an integrated public health approach: the implementation process of Greenland’s second public health program
Source: BMC Public Health. 2018 Dec 7;18:1353. doi: 10.1186/s12889-018-6253-4 (PMC6286563; doi:10.1186/s12889-018-6253-4)
Supplement: Supplementary file 1 — Table S1. “Interview guides of the conducted semi-structured interviews” provides the interview questions used in the semi-structured interviews with CHWs and HCs, which were translated verbatim from Danish to English for the purpose of publication. (PDF 134 kb) [file 12889_2018_6253_MOESM1_ESM.pdf]

Interview guides of the conducted semi-structured interviews

The interviews were conducted in Danish, and have been translated verbatim for the publication of the study in the BMC Public Health journal. The questions were slightly different in the interviews with community health workers (CHWs) in municipalities and health consultants (HCs) in the Ministry of Health respectively. Therefore, the questions of the two interview guides are here displayed next to each other.

	INTERVIEW GUIDE FOR CHWs IN MUNICIPALITIES	INTERVIEW GUIDE FOR HCs IN THE MINISTRY OF HEALTH
	**Part 0 - General information**	
1	In which municipality do you work?	Which topic area of Inuuneritta II do you work with?
2	How long have you worked in this municipality?	How long have you worked with this topic area?
3	In what position are you employed?	not included
4	How long have you been employed in that position?	How long have you been employed in that position?
5	Which department does health promotion and prevention belong to in your municipality?	not included
6	What is your educational background?	What is your educational background?
7	What are your main tasks in your daily work?	not included
8	Have you worked in other municipalities before?	Have you earlier worked in a municipality?
	**Part 1 - Inuuneritta II**	
1	Which topic areas are prioritised in your municipality in terms of health promotion?	a. When you started in your position, how did you approach the task?<br>b. Which topic areas are prioritised in the ministry, when talking about health promotion?
2	Is there a specific target group that you focus on?	Is there a specific target group that you focus on?
3	Is the municipality's health promotion work in any way based on the public health program Inuuneritta II?	In which topic area do you experience the Ministry of Health to be most active?
4	How does the municipality work with Inuuneritta II?	Have you developed an action-plan for your topic area?
5	How do you experience that the general perception of Inuuneritta II is among managers and CHWs in the municipality?	How do you experience municipalities and CHWs working with Inuuneritta II?
6	Have there been areas or recommendations in the program where you experienced difficulties regarding implementing it in your municipality?	not included

7	Which topic areas are you most active in?	In which topic area do you experience municipalities are most active?
8	Can you tell me about a specific initiative within health promotion which you work with right now? A project that you are very satisfied with.	not included
9	Now the other way around. Can you tell me about a current project within health promotion, which you experience to not work well?	not included
	**Part 2 - Collaboration**	
1	Who do you collaborate with in health promotion?	How do you experience the collaboration with other ministries regarding Inuuneritta II?
2	How do you experience the collaboration on health promotion across departments in the municipality?	How do you experience the collaboration with municipalities regarding Inuuneritta II?
3	Do you collaborate with the Ministry of Health regarding health promotion?	Have there been developed collaboration agreements on your topic area?
4	Which role does the hospital play in the health promotion work?	not included
5	In your opinion, who has the overall responsibility for the implementation of Inuuneritta II?	Who has the overall responsibility for the implementation of Inuuneritta II?
6	Do you make continuous evaluations of the local ongoing initiatives?	In Inuuneritta II it is described that evaluations and adjustments will be made annually, as well as monitoring and documentation of project development. Can you tell me how this has been done?
	**Part 3 - Future suggestions**	
1	How can the implementation and collaboration regarding Inuuneritta II in your opinion be improved?	a. How can the implementation and collaboration with other ministries regarding Inuuneritta II in your opinion be improved?<br>b. How can the implementation and collaboration with municipalities regarding Inuuneritta II in your opinion be improved?<br>c. Do you have suggestions for organisations that both municipalities and the Ministry of Health should collaborate with?
2	If you were the Minister of Health, what would you do to ensure we achieve the aims of Inuuneritta II?	If you were the Minister of Health, what would you do to ensure we achieve the aims of Inuuneritta II?
3	Are there other relevant persons you think we should interview?	Are there other relevant persons you think we should interview?
